# Metformin effect on gut microbiota: insights for HIV-related inflammation

**DOI:** 10.1186/s12981-020-00267-2

**Published:** 2020-03-10

**Authors:** Jing Ouyang, Stéphane Isnard, John Lin, Brandon Fombuena, André Marette, Bertrand Routy, Yaokai Chen, Jean-Pierre Routy

**Affiliations:** 1 Chongqing Public Health Medical Center, Baoyu Road 109, Shapingba District, Chongqing, China; 2 Infectious Diseases and Immunity in Global Health Program, Research Institute, McGill University Health Centre, 1001 Blvd Décarie, Montréal, QC Canada; 3 Chronic Viral Illness Service, McGill University Health Centre, 1001 Blvd Décarie, Montréal, QC Canada; 4 Department of Microbiology and Immunology, McGill University, 845 Sherbrooke Street West, Montréal, QC Canada; 5 Department of Medicine, Faculty of Medicine, Cardiology Axis of the Québec Heart and Lung Institute, Laval University, 2325 Rue de l’Université, Laval, QC Canada; 6 Institute of Nutrition and Functional Foods, Laval University, 2325 Rue de l’Université, Laval, QC Canada; 7 Research Centre for the University of Montréal (CRCHUM), 900 St Denis St, Montréal, QC Canada; 8 Hematology-Oncology Division, Department of Medicine, University of Montreal Healthcare Centre (CHUM), 1051 Rue Sanguinet, Montréal, QC Canada; 9 Division of Hematology, McGill University Health Centre, 1001 Blvd Décarie, Montréal, QC Canada

**Keywords:** Metformin, Microbiota, Gut permeability, Inflammation, HIV

## Abstract

The gut microbiota is emerging as a prominent player in maintaining health through several metabolic and immune pathways. Dysregulation of gut microbiota composition, also known as dysbiosis, is involved in the clinical outcome of diabetes, inflammatory bowel diseases, cancer, aging and HIV infection. Gut dysbiosis and inflammation persist in people living with HIV (PLWH) despite receiving antiretroviral therapy, further contributing to non-AIDS comorbidities. Metformin, a widely used antidiabetic agent, has been found to benefit microbiota composition, promote gut barrier integrity and reduce inflammation in human and animal models of diabetes. Inspired by the effect of metformin on diabetes-related gut dysbiosis, we herein critically review the relevance of metformin to control inflammation in PLWH. Metformin may improve gut microbiota composition, in turn reducing inflammation and risk of non-AIDS comorbidities. This review will pave the way towards innovative strategies to counteract dysregulated microbiota and improve the lives of PLWH.

## Introduction

The total human body hosts over 10^14^ microbes, of which around 99% are present in the gastrointestinal (GI) tract [1]. The GI microbiota encompasses thousands of bacteria, fungi, archaea, viruses and eukaryotic microbes. Bacteria make up the greatest proportion of microbes in the GI tract and are therefore most frequently studied. In addition to supporting nutrient absorption, the GI microbiota has an important role in homeostasis by preventing pathogens from entering the mucosa. Accordingly, a breakdown in the balance between “protective” *versus* “harmful” intestinal bacteria, a concept termed dysbiosis [2], can lead to barrier dysfunction and intestinal homeostasis disruption through translocation of microbial products leading to inflammation [3]. Increasing evidence has put a spotlight on the contribution of gut dysbiosis and its related inflammation in diabetes, inflammatory bowel diseases, cancer, aging and HIV infection [4–7]. Furthermore, people with type 2 diabetes mellitus (DM2) or HIV infection share comorbidities such as dyslipidemia, cardiovascular disease, depression and cancer in part through gut microbiome-mediated inflammation [8, 9].

HIV infection is characterized by a rapid decline in mucosal CD4^+^ T cell count, epithelial gut damage, translocation of microbial products into the systemic circulation and immune activation [10]. By suppressing host immune function, HIV leads to microbial dysbiosis and translocation, further contributing to chronic inflammation and immune activation [10]. Antiretroviral therapy (ART) has transformed care, leading to major improvements in the health of people living with HIV (PLWH). However, despite controlling viral load and CD4^+^ T-cell count, long-term ART reduces but does not normalize inflammation and immune activation compared to healthy people [11]. Gut barrier dysfunction persists, allowing microbial products to enter the circulation [12]. This heightened inflammation has been associated with non-AIDS comorbidities including dyslipidemia, cardiovascular disease, depression and cancer [13]. Given the close interaction between the intestinal microbiota and HIV-related inflammation, improving gut health by targeted therapies may reduce comorbidities and constitutes the topic of this review.

Isolated in the 1920s from French lilac, metformin (dimethylbiguanide) is the most commonly used drug to treat DM2. This drug acts as an anti-diabetic agent that promotes euglycemia without inducing hypoglycaemia and has few side effects. Compared with other classes of anti-diabetic drugs such as sulfonylureas or insulin, metformin use might have an anti-inflammatory effect as its use is associated with a lower risk of cardiovascular disease [14, 15]. More recently, metformin has been shown to be also beneficial in non-diabetic subjects, by reducing inflammation and aging biomarkers [16]. Metformin was reported to extend lifespan in some animal models, acting as a diet mimetic agent [17, 18]. In women with polycystic ovary syndrome, metformin decreased infertility rate while lowering markers of inflammation such as IL-6, TNF-α and intracellular adhesion molecule-1 (ICAM-1) [19]. Remarkably, Arrieta et al. showed that metformin, when combined with epidermal growth factor receptor-tyrosine kinase inhibitor (EGFR-TKI) therapy, improved survival in a randomized study for patients with advanced lung adenocarcinoma compared to EGFR-TKIs alone [20]. Aside from cancer (recently reviewed by Klil-Drori et al. [21]), multiple clinical trials are ongoing in non-diabetic individuals with different conditions using metformin as an immunometabolic drug (Table 1).

**Table 1:** Ongoing clinical trials in non-diabetic individuals using metformin

Conditions	Number of participants	Country	Clinical trial number
Cardiovascular			
Abdominal aortic aneurysm	170	Austria	NCT03507413
Hypertension obesity	360	China	NCT00538486
Coronary artery disease	200	USA	NCT00343395
Coronary artery disease	173	UK	NCT00723307
Myocardial infarction	380	Netherland	NCT01217307
Ischemic heart disease	120	China	NCT01879293
Aging			
Surgical outcomes in people over 60 y.o.	2000	USA	NCT03861767
Age-related macular degeneration	186	USA	NCT02684578
Pre-frail elderly	150	Indonesia	NCT02325245
Other conditions			
Familial adenomatous polyposis	100	Korea	NCT01725490
Nonalcoholic fatty liver disease (NAFLD)	150	Italy	NCT01544751
Chronic kidney diseases	385	Belgium	NCT03831464
Beta thalassemia major anemia	60	Egypt	NCT02984475
Chronic viral infection			
HIV infection	22	Canada	NCT02659306

Gut dysbiosis, increased gut permeability, chronic inflammation and systemic immune activation are common features of PLWH or DM2 [22–24]. Common microbiota composition changes such as decreased abundance of *Bifidobacterium, Bacteroides* and *Akkermansia* were found in DM2 and PLWH [7, 25, 26]. In some studies, metformin has been shown to positively influence GI microbiota composition and promote GI barrier integrity, resulting in reduced inflammation [27–33]. Given the benefits of metformin use in non-diabetic subjects and its well-documented effect on the composition of gut microbiota in DM2, we hypothesize that metformin lowers risk of non-AIDS comorbidities in ART-treated PLWH. Herein, we review and discuss advances in understanding the effects of metformin on gut dysbiosis and its potential applications in management of HIV-related inflammation, to reduce the risk of inflammatory non-AIDS comorbidities.

## Microbiota dysbiosis in obesity and DM2

DM2 is an increasing public health issue arising from genetic factors, sedentary lifestyle, Western diet and excessive visceral fat. First noted in 2008, alterations of gut microbiota composition in DM2 individuals have been well studied and reviewed [4, 25, 34–38]. Among the commonly reported findings, the genera of *Bifidobacterium, Bacteroides, Faecalibacterium, Akkermansia* and *Roseburia* abundance were decreased in DM2, while the genera of *Ruminococcus, Fusobacterium*, and *Blautia* were increased in DM2 [25]. It still remains unclear whether the DM2-associated dysbiosis is a cause or a consequence of glucose intake and/or regulation. In DM2 individuals, dysbiosis fosters bacterial translocation through the damaged epithelial gut barrier, leading to systemic immune activation. Bacterial lipopolysaccharide (LPS) binds to TLR4 and activates monocytes/macrophages leading to pro-inflammatory IL-6 and TNF secretion, and insulin resistance by inhibiting the insulin tyrosine kinase receptor signalling [39]. Moreover, LPS-induced inflammatory response reduced insulin-receptor signaling and glucose transport in human muscle cells [40]. In addition, DM2 patients have lower levels of short chain fatty acids (SCFAs), especially propionate and butyrate, in their feces compared with non-diabetic subjects [41]. SCFAs are a subset of fatty acids produced by the gut microbiota during the fermentation of polysaccharides, among which, anti-inflammatory acetate, propionate and butyrate are the most abundant [42, 43]. As the primary energy source for colonic epithelial cells, SCFAs improve intestinal barrier function, prevent microbial translocation and further reduce inflammation [42, 44]. Therefore, SCFAs and SCFA-producing bacteria are crucial in dampening inflammation.

Based on the association between microbiota and DM2, several groups have tried to modulate gut dysbiosis with prebiotics, probiotics and fecal microbiota transplantation (FMT) to improve insulin sensitivity in animals and humans [45–47]. Vrieze et al. conducted two studies in 2012 showing that FMT from lean controls improved insulin sensitivity in participants with metabolic syndrome, in association with increased intestinal abundance of butyrate-producing bacteria when compared with the control group receiving autologous FMT [45, 46]. Everard et al. reported that the abundance of *Akkermansia muciniphila,* a gut-protective bacterium, was 3300-fold lower in obese mice than in their lean littermates. Encouragingly, a 4-week oral gavage of live *A. muciniphila* in mice reversed high-fat diet-induced metabolic disorders [47]. In 2019, *A muciniphila* supplementation in obese people improved insulin sensitivity and reduced cholesterol levels in the absence of toxicity [48]. These studies demonstrate the implication of a disturbed gut microbiota in obesity and DM2 outcomes.

## More than meets the eye: metformin and gut microbiota modification in DM2

Among different anti-diabetic medications, metformin has been shown to profoundly alter the gut microbiota composition. Metformin decreases insulin resistance in DM2 via AMPK stimulation, reducing hepatic gluconeogenesis through modulation of several intracellular pathways [49]. However, growing evidence suggests that the effects of metformin are also mediated through changes in gut microbiota composition, an effect conserved from the nematode *Caenorhabditis elegans* to humans [17]. Metformin is predominantly concentrated in the jejunum with levels 30–300 times higher than in plasma [50]. Sum et al. showed in 1992 that intravenous administration of metformin did not improve blood glucose in contrast with oral administration in humans [51]. Moreover, depleting the microbiota using broad-spectrum antibiotics abrogated the anti-diabetic effects of metformin in high-fat diet (HFD) mice [52].

Microbiota compositional changes associated with metformin use in DM2 or healthy people are summarized in Table 2. Factors such as study population, sequencing method, dietary intake and medication may explain discrepancies between studies. However, increased *A. muciniphila* and *Lactobacillus,* and decreased *Intestinibacter* abundance were observed after metformin therapy in three studies [27, 29, 52]. *A. muciniphila* is a commensal anaerobic mucin-degrading bacterium whose abundance is positively associated with glucose regulation [52, 53]. This bacterium represents 1–5% of all intestinal bacteria in healthy individuals and has been shown to reduce insulin resistance following treatment with prebiotic polyphenols in animal models of obesity [54]. Metformin also increased abundance of *A. muciniphila* in HFD-fed mice [52]. Similarly, blood SCFA butyrate and propionate levels were shown to be increased in metformin-treated DM2 subjects due to microbiota modification [27]. Moreover, metformin treatment was shown to decrease the frequency of pathogenic Th17 cells and increase the frequency of regulatory T cells (Tregs), thus reducing inflammation in diabetes or IBD murine models [55, 56]. Gut dysbiosis and low SCFA production were associated with lower frequency of mucosal Tregs in mice and humans [57]. Bhaskaran et al. demonstrated that Tregs were essential in the anti-inflammatory effect of gut-derived SCFA in mice [58].

**Table 2:** Microbiota compositional changes associated with metformin use in DM2 or healthy people

Study, year (Country)	Participants with DM vs controls (n)	Increased bacterial abundance	Decreased bacterial abundance	Increased metabolites
People with DM2				
Karlsson 2013 [36] (Sweden)	20 *vs* 33	*Clostridium*	NA	NA
Forslund 2015 [27] (Denmark)	93 *vs* 106	*A. muciniphila, Escherichia, Lactobacillus, Roseburia, Subdoligranulum, Clostridiales*	*Intestinibacter*	Butyrate and propionate pathway expression
Cuesta-Zuluaga 2017 [53] (Colombia)	14 *vs* 14	*A. muciniphila, Butyrivibrio, B. bifidum, Prevotella*	NA	NA
Wu 2017 [29] (Spain)	22 *vs* 18	*Escherichia, Bifidobacterium, A. muciniphila*	*Intestinibacter*	Propionate, butyrate, and acetate
Sun 2018 [65] (China)	22, prospective study		*B. fragilis, B. finegoldii*	Bile acid glycoursodeoxycholic acid
Zhang 2019 [66] (China)	51 *vs* 26	*Spirochaete, Turicibacter, Fusobacterium*		Taurine and hypotaurine metabolism
Healthy, non-diabetic people				
Elbere 2018 [59] (Latvia)	18, prospective study	*Streptococcus, Enterobacteriaceae, A. muciniphila, Ruminococcacea, Blautia*	*Ruminiclostridium*	NA
Bryrup 2019 [60] (Denmark)	27, prospective study	*Escherichia/Shigella, Bilophila, Lachnoclostridium, Caproiciproducens*	*Intestinibacter, Clostridium,* *Terrisporobacter*	NA

*N/A* not available

Nevertheless, metformin increased *Escherichia* abundance which is associated with bloating and diarrhea, contributing to discontinuation of metformin in up to 30% of diabetic people [27, 29, 59, 60]. Toxicity including gastrointestinal upset, hyperlactatemia and metabolic acidosis, occurs infrequently when metformin accumulates due renal insufficiency or overdose [61]. Some cases of lactic acidosis and ketoacidosis have been reported in metformin-treated diabetic PLWH receiving stavudine (d4T) and didanosine (ddI) nucleoside analogs, no longer used in current practice [62–64]. Thus, use of metformin may contribute to risks including gastrointestinal distress and drug interactions in certain antiretroviral therapies, however, the benefits outweigh the risks.

## Microbiota, gut permeability and inflammation in HIV infection

During acute HIV infection, the virus rapidly disseminates while establishing a pool of latently infected cells [67]. The GI tract is critical for the pathogenesis of HIV infection and serves as a major site of viral replication [68]. Up to 70% of GI and 20% of peripheral blood CD4^+^ T-cells express CCR5, a chemokine receptor that serves as co-receptor allowing for the entry of HIV [67]. Thus, intestinal CD4^+^ T-cells are a preferential target of the virus and are massively depleted during early infection. In simian immunodeficiency virus (SIV) infected macaques and HIV infected humanized mice, damages to the intestinal epithelium were linked to microbial translocation [69–71]. In PLWH, the disruption in gut homeostasis also results in increased permeability of the gut and translocation of microbial products such as LPS, bacterial DNA, and fungal β-d-Glucan into the circulatory system, promoting chronic immune activation and disease progression [10, 72].

In parallel, bacterial communities found in the intestine of HIV-infected individuals have been shown to differ from those of individuals not infected with HIV independently of age, sex and sexual practice, recently reviewed by Vujkovic-Cvijin et al. [7]. Vujkovic-Cvijin et al. [73] used high-resolution bacterial community profiling and identified a dysbiotic mucosal-adherent community in HIV-infected subjects with high *Proteobacteria* and low *Bacteroidia* associated with markers of mucosal immune disruption, T-cell activation, and chronic inflammation. Rocafort et al. [74] found that ART-naïve HIV-1-infected subjects were significantly depleted in *Akkermansia, Anaerovibrio, Bifidobacterium*, and *Clostridium*, compared to HIV negative individuals. ART exposure was not associated with changes in abundance of such genera, compared with ART-naïve. In SIV infected macaques, gut dysbiosis was also observed and strongly correlated with cytokine gene expression in the gut‐draining mesenteric lymph nodes including IL-10 and IL-6 [75]. Probiotic/prebiotic supplementation improves gastrointestinal immune function, increases reconstitution and decreases inflammation in ART-treated SIV-infected macaques [76].

Accumulating evidence has shown that dysregulation of the gut microbiota metabolism plays a role in HIV disease progression. The activity of the indoleamine-2,3-dioxygenase-1 (IDO-1), an enzyme catabolising the essential amino-acid tryptophan into immunosuppressive kynurenines, has been recognized as a key factor of HIV immune dysfunction and damage to the gut mucosa [73, 77]. The activity of IDO-1 correlates with Th17 cell loss, Tregs elevation, gut and systemic inflammation, reservoir size and disease progression in HIV-infected subjects [78, 79]. Furthermore, gut dysbiosis correlated with plasma kynurenine levels in ART-treated PLWH [73]. In addition, a decreased abundance of bacteria producing the gut epithelial protector butyrate, including *Roseburia, Coprococcus, Faecalibacterium*, and *Eubacterium*, was observed in both HIV-treated and ART-naïve individuals, in association with altered SCFAs profiles [80, 81]. Finally, HIV infection is associated with increased risk of coronary heart disease beyond that explained by traditional risk factors, and altered gut microbiota has been proposed as a key contributing determinant [82]. Higher activity of the kynurenine pathway and higher trimethylamine N-oxide (TMAO) plasma levels were also associated with an increased risk of cardiovascular disease [83, 84]. TMAO is converted in the liver from trimethylamine (TMA) which is an organic compound synthesized exclusively by the gut microbiota from dietary nutrients. Haissman et al. [85] reported that microbiota-dependent TMAO levels are also associated with monocyte activation in untreated PLWH. By comparing PLWH with and without coronary heart disease, Kehrmann et al. [86] showed that high circulating TMAO was a marker of coronary heart disease in association with the fecal abundance of *Phascolarctobacterium, Desulfovibrio, Sutterella*, and *Faecalibacterium*.

## Insights on the use of metformin in non-diabetic PLWH

**Fig. 1:**
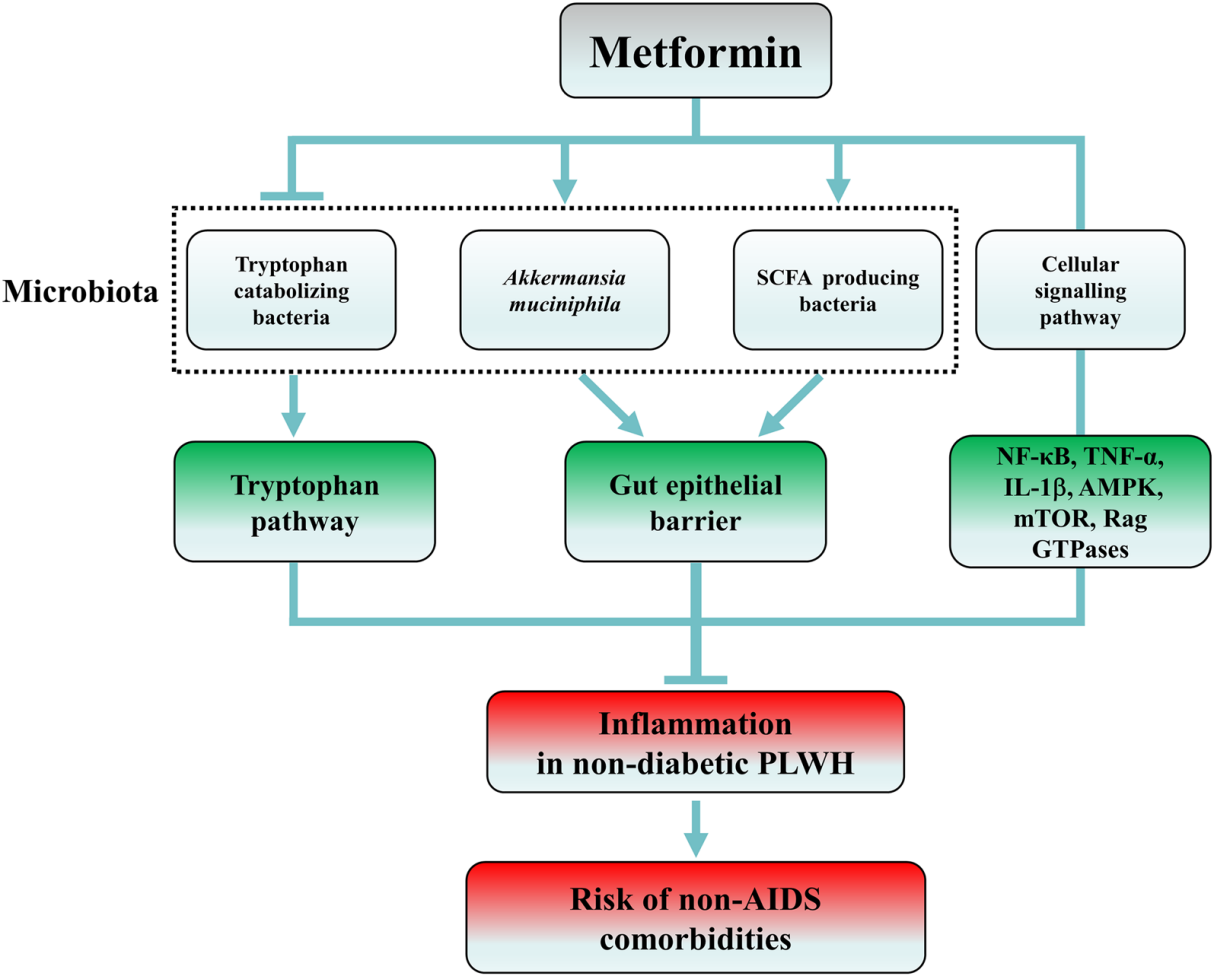
Potential effects of metformin in non-diabetic PLWH

Treatment with metformin in PLWH has been shown to decrease lipodystrophy syndrome, hyperlipidemia and insulin sensitivity [87–90]. Moreover, Fitch et al. reported that metformin prevented progression of coronary artery calcification (CAC) and calcified plaque volume in PLWH with metabolic syndrome [90]. Shikuma et al. recently reported that metformin reduced CD4^+^ T‐cell exhaustion in non-diabetic ART‐treated PLWH [91]. Our team is currently carrying out a pilot study to determine the effect of metformin in non-diabetic ART-treated PLWH (NTC02659306) [92]. Metformin might be a promising treatment to control inflammation in non-diabetic PLWH through multiple pathways illustrated in Fig. 1.

As several studies showed that metformin increased *A. muciniphila* abundance in diet-induced obese mice and DM2 patients [27, 29, 52, 53, 93], metformin may also increase *A. muciniphila* abundance in ART-treated PLWH to promote epithelial barrier integrity and decrease inflammation. Indeed, Reunanen et al. showed that *A. muciniphila* improved gut barrier integrity by binding to enterocytes in Caco-2 and HT-29 human colonic cell models [94]. Moreover, *A. muciniphila* administration reduced translocation of bacterial LPS and adipose tissue inflammation in an obese insulin-resistant mouse model [47].

In PLWH, there is a lower abundance of butyrate-producing bacteria in the gut microbiota [80, 81]. Interestingly, metformin was shown to increase the abundance of butyrate producing bacteria in both diabetic and healthy individuals [27, 60]. We therefore suggest that metformin, through increasing butyrate production, may decrease inflammation in ART-treated PLWH by enhancing intestinal epithelial barrier function, preventing microbial translocation and increasing mucosal Treg frequency [44, 58, 81].

Tryptophan catabolism and the kynurenine pathway were also associated with disease progression and HIV reservoir size in ART-treated PLWH [78]. Moreover, dysbiosis was associated with the kynurenine pathway in PLWH [73]. As Muzik et al. reported that metformin treatment of insulin resistant diabetic subjects was associated with down-regulation of the kynurenine pathway [95], metformin might also decrease tryptophan catabolism in non-diabetic ART-treated PLWH by altering microbiota composition.

Metformin may also reduce HIV-related inflammation independently of microbiota modification by modulating several signalling pathways: (1) suppressing nuclear factor κB activation, which enhances HIV transcription and induces the expression of various pro-inflammatory genes; (2) indirectly reducing secretion of proinflammatory cytokines such as tumour necrosis factor-alpha (TNF-α) and interleukin-1-β (IL-1β), which remain a high level in PLWH; (3) inhibiting mTOR, through an AMPK-dependent mechanism, reducing CD4 T cell activation, in turn reducing inflammation; (4) indirectly blocking mTOR signalling by inhibiting Rag GTPases [96–98].

## Conclusions

Gut dysbiosis has been associated with DM2- and HIV-related gut permeability, microbial translocation and inflammation. Metformin has been shown to modulate gut microbiota composition in diabetic and non-diabetic people, in association with reduction of gut damage and inflammation. However, the efficacy and safety of metformin to control inflammation and reduce risk of inflammatory comorbidities in non-diabetic PLWH are still unknown. Direct evidence is needed to verify and endorse the beneficial effects of metformin as a possible modulator of HIV-related inflammation. Following our pilot study, larger randomized placebo-controlled studies will be needed to assess the independent effect of metformin on gut dysbiosis and inflammation in non-diabetic PLWH. Collaborative effort encompassing microbiology, clinical care, epidemiology and artificial intelligence will define the dose and duration to obtain the optimal benefit of metformin as an immune modulator in ART-treated PLWH.

## Data Availability

Not applicable.

## Abbreviations

AMPK: AMP-activated protein kinase
APC: Antigen presenting cell
ART: Antiretroviral therapy
cAMP: Cyclic adenosine monophosphate
EGFR-TKI: Epidermal growth factor receptor-tyrosine kinase inhibitor
FMT: Fecal microbiota transplantation
GI: Gastrointestinal
HFD: High-fat diet
IDO-1: Indoleamine-2,3-dioxygenase-1
ICAM-1: Intracellular adhesion molecule-1
LPS: Lipopolysaccharide
MGWAS: Metagenome-wide association study
NAFLD: Nonalcoholic fatty liver disease
PLWH: People living with HIV
PKA: Protein kinase A
Tregs: Regulatory T cells
SCFA: Short chain fatty acid
SIV: Simian immunodeficiency virus
TLR-4: Toll-like receptor
TMA: Trimethylamine
TMAO: Trimethylamine N-oxide
IL-1β: Tumour necrosis factor-alpha (TNF-α) and interleukin-1-β
DM2: Type 2 diabetes mellitus
